# Simulating Photodissociation in Strong Field Enabled by the Random Phase Thermal Wavefunction Approach

**DOI:** 10.1021/acs.jpca.6c05555

**Published:** 2026-09-11

**Authors:** Bar Ezra, Shimshon Kallush, Ronnie Kosloff

**Affiliations:** † The Institute of Chemistry and the Fritz Haber Centre for Theoretical Chemistry, 26742 The Hebrew University of Jerusalem, Jerusalem 9190401, Israel; ‡ 99055 H.I.T. - Holon Institute of Technology, 52 Golomb St., Holon 58102, Israel

## Abstract

Simulating photodissociation becomes challenging when the number of states involved grows large. The computational complexity is usually reduced by restrictions that limit the model’s scope. We present a first-principles quantum model for strong-field photodissociation that treats rotational dynamics explicitly. The random-phase thermal wavefunction method reduces the large computational cost. The simulation yields experimental observables, such as the momentum angular distribution of the photofragments. We study how these observables converge at two field intensities, with each set at a different temperature. The method is designed to reach conditions where exact numerical methods become intractable; here, we validate it against exact results in the regime where both are available. Beyond the conventional dependence on system size and number of realizations, we find that convergence depends on the observable’s sensitivity to coherence.

## Graphical abstract

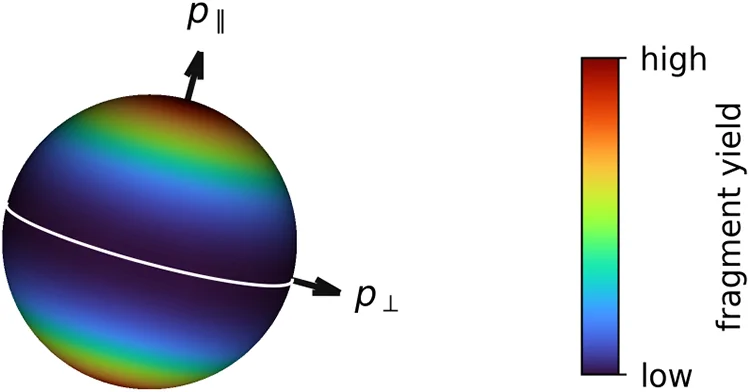

## Introduction

Time-domain spectroscopy can be directly modeled by solving the time-dependent Schrödinger equation. Direct modeling for a strong-field photodissociation process has almost no alternative, since analytical methods are not applicable and perturbation expansions do not converge. This work presents a first-principle simulation method applicable to the photodissociation process of a diatomic molecule. The simulation is guided by strong-field pulsed experiments. The goal is that the simulation outcome could be directly comparable to experimental observables, specifically to the velocity distribution of the photofragments. The simulation is first-principles in structure: given the Hamiltonian, initial temperature, charge, and mass, it propagates the dynamics with no fitted dynamical parameters.

The knowledge of the total Hamiltonian and initial state is required to solve the time-dependent Schrödinger equation. The Born–Oppenheimer decomposition allows us to separate the electronic and the nuclear terms.^1^ Ab initio electronic structure methods are able to accurately calculate the electronic potential surfaces and the transition dipole moments associated with the photodissociation. The present study concentrates on solving the nuclear dynamics, specifically the dynamics of the vibrational and rotational degrees of freedom of a diatomic molecule on multiple electronic surfaces. The strong coupling between the molecular degrees of freedom opens multiple channels to unbound electronic states where the molecule dissociates.

Within the common experimental framework,^2,3^ various physical measures of the photofragments are observed far from the dissociation point. The simulation should be able to calculate the same observable and specifically the angular distribution of the outgoing momentum. Such a calculation is set in a large phase-space volume, which translates to a very large computational Hilbert space. In the weak-field regime, one can use perturbation theory, and the calculation is simplified significantly since selection rules limit the accessible Hilbert space. In strong-field photodissociation processes, many interference channels become open. As a result, the calculation must include all of the relevant quantum states that participate in the dynamical process.

Solving the time-dependent Schrödinger equation for a large Hilbert space is a difficult task. The computation effort scales with the size of the Hilbert space, the product of the energy range, and time period. The Hilbert space size is determined by the phase-space volume containing the problem. The phase-space is restricted by the maximum energy the molecule can carry and by the asymptotic distance estimated by the effective dissociation point. The number of angular-momentum states required for the calculation is determined by the initial temperature of the molecule and the strength of the radiation field. Each participating photon changes the rotational angular momentum by one unit of ℏ. Therefore, when the molecule is subjected to a strong radiation field, the size of the manifold of the accessible states increases significantly. For a photodissociation study, the Hilbert space is vast, of the order 10^6^ states for the representation of the whole ro-vibronic manifold (where, the vibration is represented on a grid of size (∼10^3^), rotational states (∼ 10^2^) and spin-orbital states (∼ 10^1^)). In principle, the initial state is a thermal mixed state formally requiring a density-operator description. The dimension of the density operator is the square of the Hilbert space; this means that such a calculation is prohibitively expensive.

Several studies have addressed the computational complexity of similar systems. In the study of *I*_2_^–^, one of the most studied diatomic molecules, the complexity is reduced by neglecting the rotational degree of freedom due to the heavy molecule.^4–6^ For the *H*_2_^–^ case, the lightest molecule, the study was limited to low initial temperatures, resulting in only the ground ro-vibrational state.^7–9^ A commonly employed approximation assumes that the initial orientation is fixed to the perpendicular direction of the polarization of the laser field. This leads to a significant reduction of the accessible Hilbert space (practically to *M* = 0 only)^10^ with the price that the simulation scope is limited.

The random-phase wavefunction (RPW) method is employed in this study to overcome the challenge of the large Hilbert space. It replaces a density-operator calculations by a finite sample of random-phase thermal wavefunctions.^11,12^ Previously, the method has been used to calculate the dynamics of molecular systems including rotational states.^13–17^ The present study aims to develop methods to calculate the full asymptotic photofragment distribution. This requires studying the convergence of the calculation with respect to the number of RPWs employed. Typically in the RPW theory, the convergence of the observables scales with the number of realizations, *K*, and the Hilbert size, *N*_S_, as $∼\frac{1}{\sqrt{K}}\cdot \frac{1}{\sqrt{N_{S}}}$ .^13^ We investigate how many realizations *K* each observable needs to reach a target accuracy. We find this number depends strongly on the observable’s sensitivity to coherence.

The developed simulation is general, it can be used to calculate a variety of processes and systems. We use the *F*_2_^–^ properties to enable later comparison with the experiments of Shahi, Albeck, and Strasser.^2,3^ Note, however, that the goal of the present work is to study the random-phase method and for this task we simplified the system and employed only two of the four potentials (see below).

## Methods

The dynamics of a gas phase diatomic molecule takes place on *n* electronic surfaces and three ro-vibrational internuclear degrees of freedom, the internuclear distance, and the two rotational angles. The system Hamiltonian includes the nuclear Hamiltonian of all electronic states, and the radiation coupling elements between the states are mediated by the transition dipole moment. In the Born–Oppenheimer decomposition,^1^ the Hamiltonian can be written as

1 $${Ĥ}_{sys}=\sum_{n}\left({Ĥ}_{n}⊗\left|n\right\rangle \left\langle n\right|+\sum_{k\neq n}\mu_{n,k}\cdot \varepsilon ⊗\left|n\right\rangle \left\langle k\right|\right)$$

2 $${Ĥ}_{n}=\frac{{\mathrm{P̂}}_{r}^{2}}{2m_{r}}+V_{n}\left(r\right)+\frac{1}{2I}{\mathrm{R̂}}^{2}\left(\theta ,\phi \right)$$

where *Ĥ*_*n*_ is the nuclear Hamiltonian operator of the *n*th electronic state. *Ĥ*_*n*_ contains the electronic potential of the *n* electronic state, *V*_*n*_(*r*), at each internuclear distance *r*, (presented in Table 1) and the total nuclear kinetic energy. The kinetic energy is composed of the linear motion term, $\frac{{\mathrm{P̂}}_{r}^{2}}{2m_{r}}$ , and the rotational motion term, $\frac{1}{2I}{\mathrm{R̂}}^{2}\left(\theta ,\phi \right)$ , where *m*_*r*_ is the reduced mass. Here, *P*_*r*_ is the linear momentum, *I*(*r*) = *m*_*r*_ *r* ^2^ is the moment of inertia, and **R̂**(θ, ϕ) is the nuclear rotational angular-momentum operator. The laser field, ε, is a transform limited pulse with the parameters presented in Table 1.

**Table 1:** Model Parameters

parameter	value	
reduced mass	*m*_*r*_ = 1900 a.u.	
ground electronic potential	*V*_g_(*r*) = *D*·(1 – e^–*a*·(*r*–*r*_e_)^)^2^	
excited electronic potential	$V_{e}\left(r\right)=\frac{4.8}{r^{6}}$	
effective width	$a=\omega /\sqrt{(2D/m_{r}})$	
equilibrium distance	*r*_e_ = 2 bohr	
frequency	ω = 916 cm^–1^	
bond dissociation energy	*D* = 1.256 eV	
transition dipole moment	μ = 0.2 a.u.	
grid spacing	Δ*r* = 0.0244 bohr	
grid end	*r*_max_ = 50 bohr	
time step	Δ*t* = 5·10^–2^ fsec	
number of time steps	*N*_*t*_ = 7500	
pulse width	30 fsec	
central wavelength	400 nm	
intensity	10^10^ W/cm^2^	10^13^ W/cm^2^
temperature	80 K	50 K
thermally occupied *J*_max_	40	25
reachable *J*_max_	55	32

The scalar product between the transition dipole operator, **μ**_*n*,*k*_, and the radiation field, **ε**, can generate different transition schemes. The specific scheme is influenced by the states involved, symmetries, and the field polarization. The details are elaborated in our previous work.^18^ The present study contains only two ^1^Σ singlet electronic states. Hence, under these conditions, the nuclear rotational angular-momentum operator merges into the total angular momentum of the molecule, **R̂**(θ, ϕ) = **Ĵ**(θ, ϕ). In the absence of other angular momenta, and the Hund’s case (*a*),^19^ the most common case and the one used here, the projection of ***Ĵ*** (θ, ϕ) on the laboratory axis, *Ẑ*, is *m* and on the internuclear axis is Ω.

Similar to our previous work,^18^ we expand the angles’ coordinate by the Wigner rotation matrices, *D*_*m*,Ω_^*j*^(φ, θ). With this basis set, each state is described by *r*, *j*, *m*, and Ω. The density matrix of the molecular state at time *t* is

3 $$\mathrm{\rho ̂}\left(r,r^{\prime },\theta ,\theta^{\prime },\varphi ,\varphi^{\prime },n,n^{\prime };t\right)=\sum_{\zeta ,\zeta^{\prime }}\left|n\right\rangle \left\langle n^{\prime }\right|⊗a_{n,n\prime ,\zeta ,\zeta \prime }\left(r,r^{\prime };t\right)⊗\left|\zeta \right\rangle \left\langle \zeta^{\prime }\right|$$

where the summation is over all rotational states, ζ = {*j*, *m*, Ω}, thus |ζ⟩ is the wavefunction with the quantum numbers |*j*, *m*, Ω⟩, expanded by the *D*_*m*,Ω_^*j*^(φ, θ) matrices. *a*_*n*,*n*′,ζ,ζ′_ (*r*, *r*′; *t*) is the nuclear matrix coefficients at each electronic and rotational state. This work includes only ^1^Σ states Ω = 0, and the matrices are identical to the spherical harmonics *Y*_*j*,*m*_(φ, θ). For linearly polarized external field, the coupling elements between the states, due to the interaction with the field, are proportional to cos­(θ) ∝ *D*_0,0_^1^(φ, θ).

To mimic the experimental process, a thermal ensemble of molecules is subjected to a pulsed laser source. Within the temperatures considered, the electronic and vibrational degrees of freedom are in their ground state. The initial state is a Boltzmann distribution of several rotational components and can be written as

4 $$\mathrm{\rho ̂}\left(r,r^{\prime },\theta ,\theta^{\prime },\varphi ,\varphi^{\prime },n,n^{\prime };t=0\right)=\sum_{\zeta_{i}}P_{B}\left(j_{i}\right)a_{0,0,\zeta_{i},\zeta_{i}}\left(r,r^{\prime };t=0\right)\cdot \left|0\right\rangle \left\langle 0\right|⊗{\mathrm{P̂}}_{0,j_{i}}\left(r\right)⊗\left|\zeta_{i}\right\rangle \left\langle \zeta_{i}\right|$$

where ${\mathrm{P̂}}_{0,j_{i}}\left(r\right)=\left|\Phi_{n=0,j_{i}}\left(r\right)\right\rangle \left\langle \Phi_{n=0,j_{i}}\left(r\right)\right|$ is the projection onto the ground vibrational state that conditioned by the rotational state, *j*_*i*_. $P_{B}\left(j_{i}\right)=\frac{\exp\left(-\beta E_{j_{i}}\right)}{Z}$ are the Boltzmann coefficients, where $Z=\sum_{l}\left(2l+1\right)\exp\left(-\beta E_{l}\right)$ is the partition function. Note, that the rotational energy eigenvalues that satisfy *Ĥ*_rot_ |ψ_ζ_*i*__⟩ = *E*_*j*_*i*__|ψ_ζ_*i*__⟩ depend only on the total angular momentum *j*.

The time evolution of the state is carried out by a first-principle propagation of the time-dependent Schrödinger equation: *ρ̂*(*t*) = *Û*(*t*)*ρ̂*(*t* = 0)*Û* ^†^(*t*) where $i\hbar \frac{d}{dt}Û\left(t\right)=Ĥ\left(t\right)Û\left(t\right)$ . Observables are calculated by the data provided by the density operator, *ρ̂*(*t*), so that for any operator *Â*

5 $${\left\langle Â\right\rangle }_{t}=\mathrm{tr}\left(Â\rho \left(t\right)\right)=\mathrm{tr}\left(ÂÛ\left(t\right)\rho \left(t=0\right){Û}^{†}\left(t\right)\right)$$

Under the common experimental conditions for the molecular beam considered here, the typical incoherent processes such as collisions and spontaneous decay take place on the time scales of *t* ≥ 1 nsec. Dissociation under a 30 fsec laser pulse is achieved by ∼100 fsec. The dynamics are therefore coherent. Therefore, the expectation value can be evaluated, employing the basis where the initial density operator is diagonal |ψ̃_0,0,ζ_*i*__⟩ = |ψ_0_^e^⟩ ⊗ |ψ_0_^ν^⟩ ⊗ |ζ_*i*_⟩, giving by

6 $${\left\langle Â\right\rangle }_{t}=\sum_{n,\nu ,\zeta }P_{B}\left(j_{i}\right)\left\langle {Û}^{†}\left(t\right){\mathrm{\psi ̃}}_{0,0,\zeta_{i}}\left(t=0\right)\right|Â\left|Û\left(t\right){\mathrm{\psi ̃}}_{0,0,\zeta_{i}}\left(t=0\right)\right\rangle$$

Using eq 6, we can decompose the expectation value into the expectation values of the individual components. Each component requires the solution for time-dependent Schrödinger equation: $i\frac{d}{dt}\psi =Ĥ\left(t\right)\psi$ . Numerical methods for the wavefunction propagation such as the Chebychev approximation scale as *N*_S_^2^.^20–23^ Observables that are computed by eq 6 will be used as a reference to the results that will be derived by the random-phase method.

The size of Hilbert space of linear molecules, that is the number of occupied states *N*_S_, scales linearly with the temperature. The experiments considered on *F*_2_^–^ are carried out at an estimated temperature of *T* = 100 *K*, where *N*_S_ ∼ 10^6^. We ran two cases at lower temperatures (see Table 1) to keep the exact reference computable.

The light-induced coupling between these states further complicates the problem. At low intensity, the transition can be approximated perturbatively, which keeps the scale of interacting states to *N*_S_^2^, while at high intensities, strong fields, the process requires full and exact calculation, which increases the scale of the states to *N*_S_^3^.

To overcome the challenge of the large number of states, the random-phase wavefunction method is used. An efficient representation of the state is done by the use of a limited ensemble of pure states, mimicking the initial thermal ensemble. The initial state of a single random-phase wavefunction is written as

7 $$\left|\Psi^{k}\left(r,\theta ,\varphi ;t=0\right)\right\rangle =\sum_{\zeta_{i}}e^{i\chi_{\zeta_{i}}^{k}}\sqrt{P_{B}\left(j_{i}\right)}\sqrt{{ã}_{0,\zeta_{i}}\left(t=0\right)}\left|0\right\rangle ⊗\left|\Phi_{0,\zeta_{i}}\left(r\right)\right\rangle ⊗\left|\zeta_{i}\right\rangle$$

where the phases χ_ζ_*i*__^*k*^ are chosen randomly, and *ã*_0,ζ_*i*__ (*t* = 0) are the coefficients corresponding to the diagonal elements of *a*_0,0,ζ_*i*_,ζ_*i*__(*r*, *r*; *t* = 0), and the angular part is introduced by |ζ_*i*_⟩. The propagation of the wavefunction is done by solving the time-dependent Schrödinger equation, |Ψ^*k*^(*r*, θ, φ; *t*)⟩ = *U*(*t*) |Ψ^*k*^(*r*, θ, φ; *t* = 0)⟩ (using the Chebyshev expansion^22^ and absorption boundary condition in additional auxiliary channels as fully described at our previous work^18^). For large number of realizations *k*, the set of random-phase wavefunctions is complete basis of states

8 $$\lim_{K\to \infty }\frac{1}{K}\sum_{k=1}^{K}\left|\Psi^{k}\left(r,\theta ,\varphi ;t\right)\right\rangle \left\langle \Psi^{k}\left(r^{\prime },\theta^{\prime },\varphi^{\prime };t\right)\right|=\mathrm{\rho ̂}\left(r,r^{\prime },\theta ,\theta^{\prime },\varphi ,\varphi^{\prime },n,n^{\prime };t\right)$$

The efficiency of the random-phase wavefunction method increases with the size of Hilbert space, i.e., with the number of random phases, which increases with the temperature.^13^

The time-dependent observables are computed for each random-phase wavefunction and then averaged over *K* realization. The error with respect to the exact solution, the reference, decreases with the number of realizations, *K*. The observables are calculated using the RPW as follows

9 $${\left\langle Â\right\rangle }_{t}^{K}=\frac{1}{K}\sum_{k=1}^{K}\sum_{n,\nu ,\zeta }\left\langle \Psi^{k}\left(r,\theta ,\varphi ;t\right)\right|Â\left|\Psi^{k}\left(r,\theta ,\varphi ;t\right)\right\rangle$$

In the following section, we present the results calculated by using the random-phase wavefunction method on the spatially resolved photodissociation. The quantum observables are then compared to numerically exact computations,^18^ carried out by propagating of all of the initially occupied states and thermal summation of their expectation values. For a convergence study, the exact numerical result is the appropriate reference: it isolates the error of the random-phase sampling from any error in the potentials. The comparison to experiment is a separate question, requiring the ab initio potentials discussed above.

## Results

A first-principles simulation has been designed to simulate the photodissociation process of a diatomic molecule. The RPW method has been employed to overcome the large Hilbert-space challenge. Thus, the initial state was sampled from the Boltzmann thermal state by random-phase wavefunctions, eq 7. The photofragment outcome state is represented by the final density operator composed from averaging the individual initial RPW states, eq 8.

Two opposing constraints pin the choices. The RPW method needs many occupied states to be efficient, so low intensity forces a high temperature. The exact reference must stay computable, so high-intensity forces a low temperature. Our two cases therefore differ in both intensity and temperature and do not isolate intensity by themselves. In each calculation, we compared the RPW result to the exact computation, propagating each initial state separately in the full Hilbert space.

First, the dissociation probability and the average kinetic energy of the fragments were calculated. The dissociation probability, $P^{k}$ , and the kinetic energy, $E^{k}$ , for each realization *k* from the final wavefunction are

$$P^{k}=\left\langle \Psi_{n\neq 0}^{k}\left(r,\theta ,\varphi ;t=t_{final}\right)|\Psi_{n\neq 0}^{k}\left(r,\theta ,\varphi ;t=t_{final}\right)\right\rangle$$ 10

11 $$E^{k}=\left\langle \Psi_{n\neq 0}^{k}\left(r,\theta ,\varphi ;t=t_{final}\right)\left|{\mathrm{P̂}}_{r}^{2}\right|\Psi_{n\neq 0}^{k}\left(r,\theta ,\varphi ;t=t_{final}\right)\right\rangle$$

The exact dissociation probability, calculated according to eq 10, is 9.28 × 10^–3^ at the high intensity and 6.57 × 10^–6^ at the low intensity. Figure 1 presents the deviation of the approximate computation from the exact one, for the dissociation probability P , and the fragments’ average kinetic energy *E*_*k*_.

**Figure 1:**
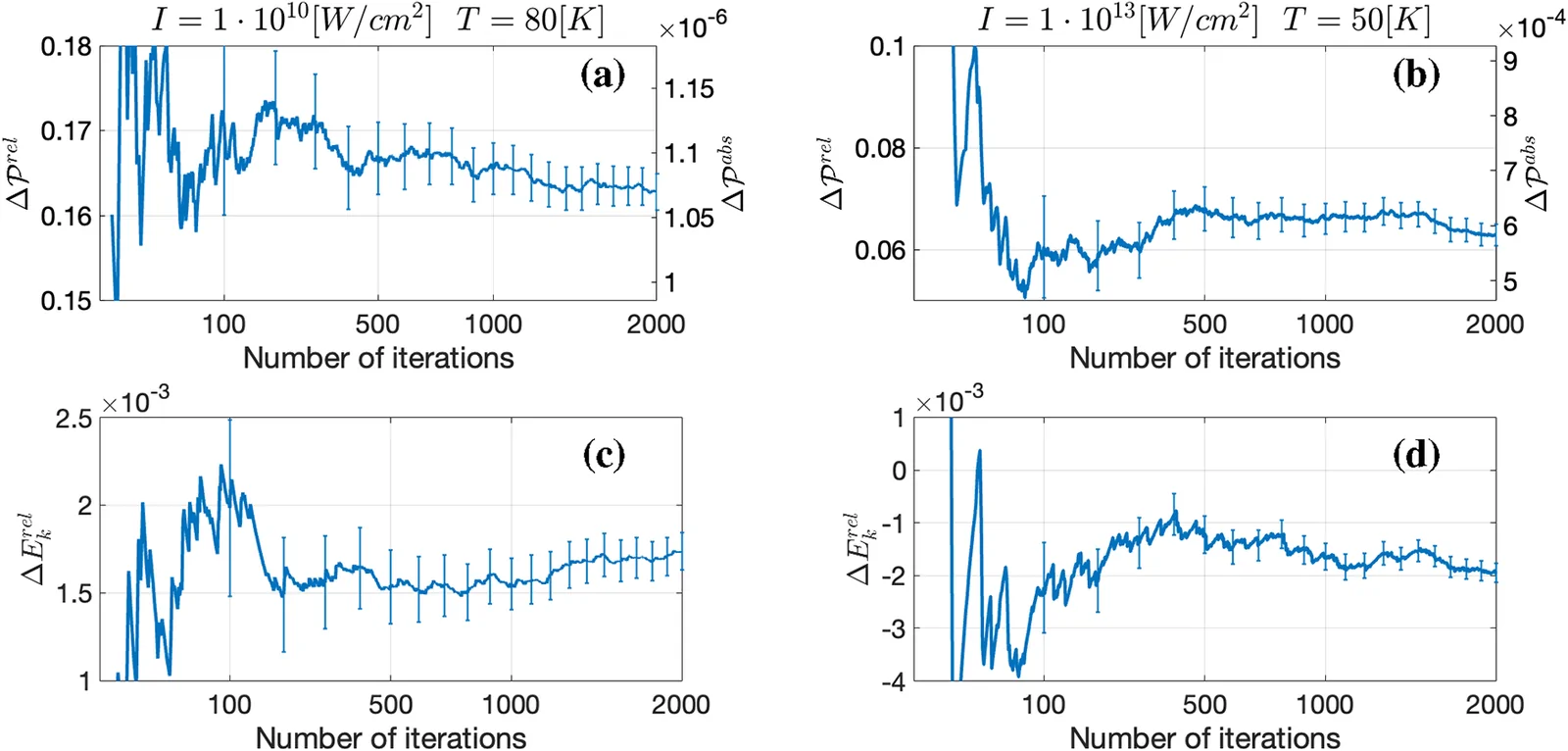
Observables errors as a function of the number of realizations: dissociation probability and kinetic energy. Results for the low intensity, left panel-figures (a, c), and high intensity, right panel-figures (b, d). Panels (a, 1b) show the error in the dissociation probability, P . The error is displayed by two axis, the absolute difference between the probabilities, the inner axis. The outer axis display the relative error. Panels (c, 1d) present the relative error in the kinetic energy as a function of the number of realizations.

The error bars correspond to the standard deviation of the results from the mean value at each given number of realizations. For any observable *A* this is given by: $\frac{1}{K}\sqrt{\sum_{k=1}^{K}(<A{>}_{k}-<A{>}_{K}{)}^{2}}$ , where <*A*>_*K*_ is the averaged value of *A* after *K* realizations and <*A*>_*k*_ is the value for a given realization *k*. The convergence of the deviation with the number of realizations is visible.

Figure 1a,b displays the error of the dissociation probability. Two scales are displayed: the relative error $\Delta P_{\mathrm{rel}}=\left|P_{\mathrm{RPW}}-P\right|/P$ in the outer axis and absolute error $\Delta P_{\mathrm{abs}}=\left|P_{\mathrm{RPW}}-P\right|$ in the inner axis. As expected, at the high intensity the large overall P leads to an absolute error much larger compared to the low intensity. The relative error of this case is, however, significantly lower than at the low intensity. Note that a comparison to the experimental output reveals that, at least for commonly used methods, the resolution for the low intensity regime is way beyond the experimental. Absolute experimental values for the dissociation probability for the higher intensity regime are even harder to be determined.

The kinetic energy of the photofragments is used experimentally to track the asymptote of the electronic state from which the dissociation takes place. Panels (c) and (d) in Figure 1 present the average kinetic energy relative error. The error at both intensities was already lower than 1% after 200 realizations.

Figure 2 presents the angular distribution of the momentum fragments calculated by the direct method, Figure 2a,2b, and the RPW method, Figure 2c,d. The angular distribution of the outgoing momentum, *P*, is calculated as follows

$$D^{k}\left(P,\theta \right)=\int d\phi \underset{m,\Omega ,n>0}{\sum }{\left|\underset{j}{\sum }\underset{\zeta_{i}}{\sum }e^{i\chi_{\zeta_{i}}^{k}}\sqrt{P_{B}\left(j_{i}\right)}\sqrt{{ã}_{n,0,\zeta ,\zeta_{i}}\left(t=t_{final}\right)}\cdot D_{m,\Omega }^{j}\left(\theta ,\phi \right)\right|}^{2}$$ 12

where *ã*_*n*,0,ζ,ζ_*i*__(*t* = *t*_final_) are the coefficients in momentum representation corresponding to the *a*_*n*,0,ζ,ζ_*i*__(*t* = *t*_final_) coefficients. Note that the summation over *j* is a quantum summation that conserves the phases while the summations over *m* and Ω are classical. The difference is an outcome of the dynamics, which generates coherences only between different values of *j* with the same values of *m* and Ω.

**Figure 2:**
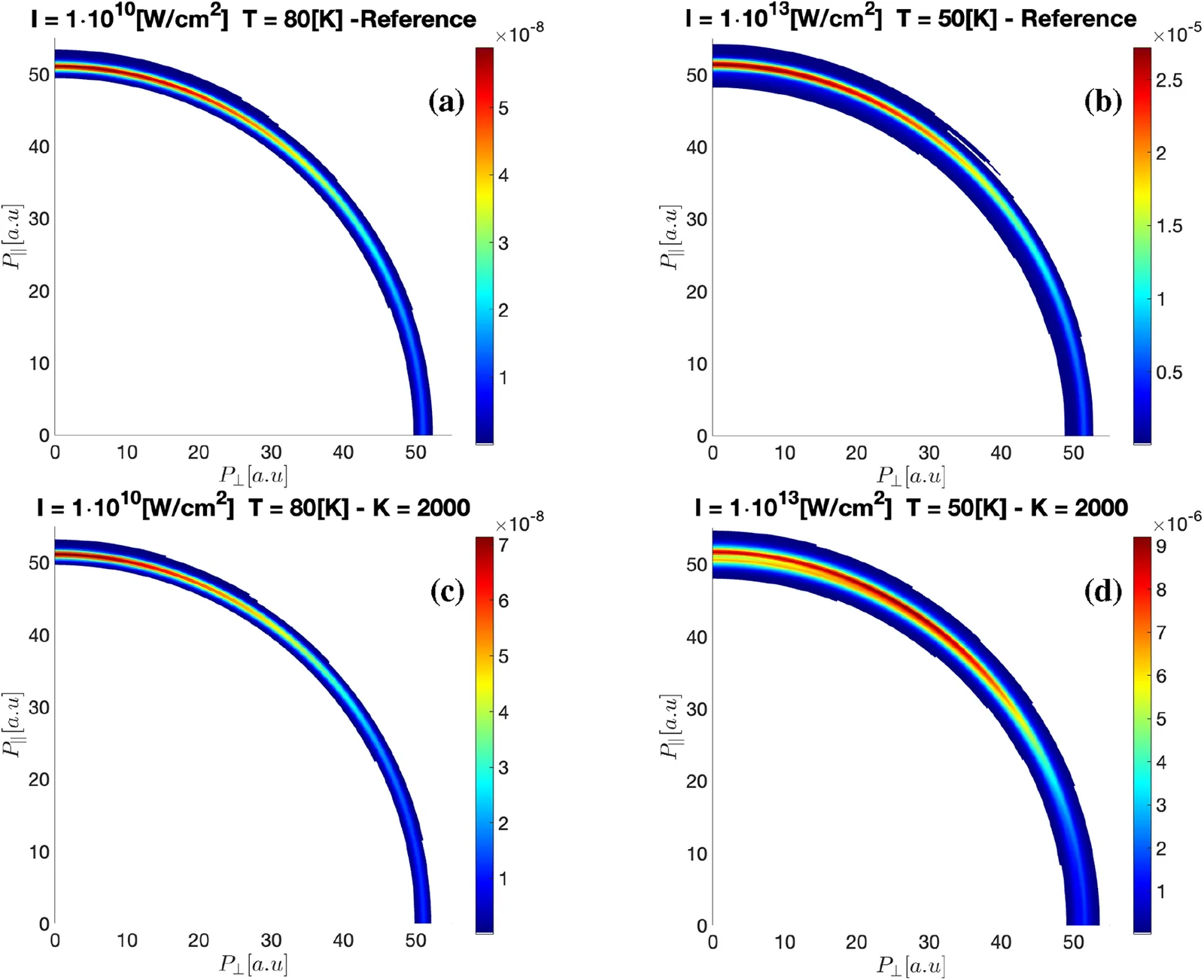
Angular distribution of the outgoing momentum, *P*, shown as a density plot. Exact (upper panels (a, b)) and RPW method after 2000 realizations (bottom panels (c, d)). Left and Right panels are for low and high intensities, respectively.

The two distributions are qualitatively similar with the same central momentum peaks and parallel orientation. However, changes in small details could be observed, especially when comparing the distributions at high-intensity panels, Figure 2d,2b.

A more detailed analysis is obtained by comparing separately the directions in the azimuth and the absolute value of the momentum. The upper panels in Figure 3 presents the distribution as a function of the momentum for *P*_⊥_ = 0. Note, that only a very narrow range of values from the full momentum grid are plotted. The lower panel at Figure 3 presents the distribution as a function of the angle θ, taken at the most probable momentum. At low intensity, the convergence of both distributions to the exact distribution is fast, while at high laser intensity, the distribution has not converged even after 2000 realizations. In particular, the momentum-magnitude peak at high intensity reaches only about a third of the exact height and barely changes between 100 and 2000 realizations. Strong interference at high intensity builds high coherence into this observable, which drives the slow convergence. Specifically, we see the sensitivity to interference on the momentum and angular distribution. On top of this, one should beware of the fact that the grid resolution at the *P* axis is poor, due to the very narrow width of the distribution in the momentum space.

**Figure 3:**
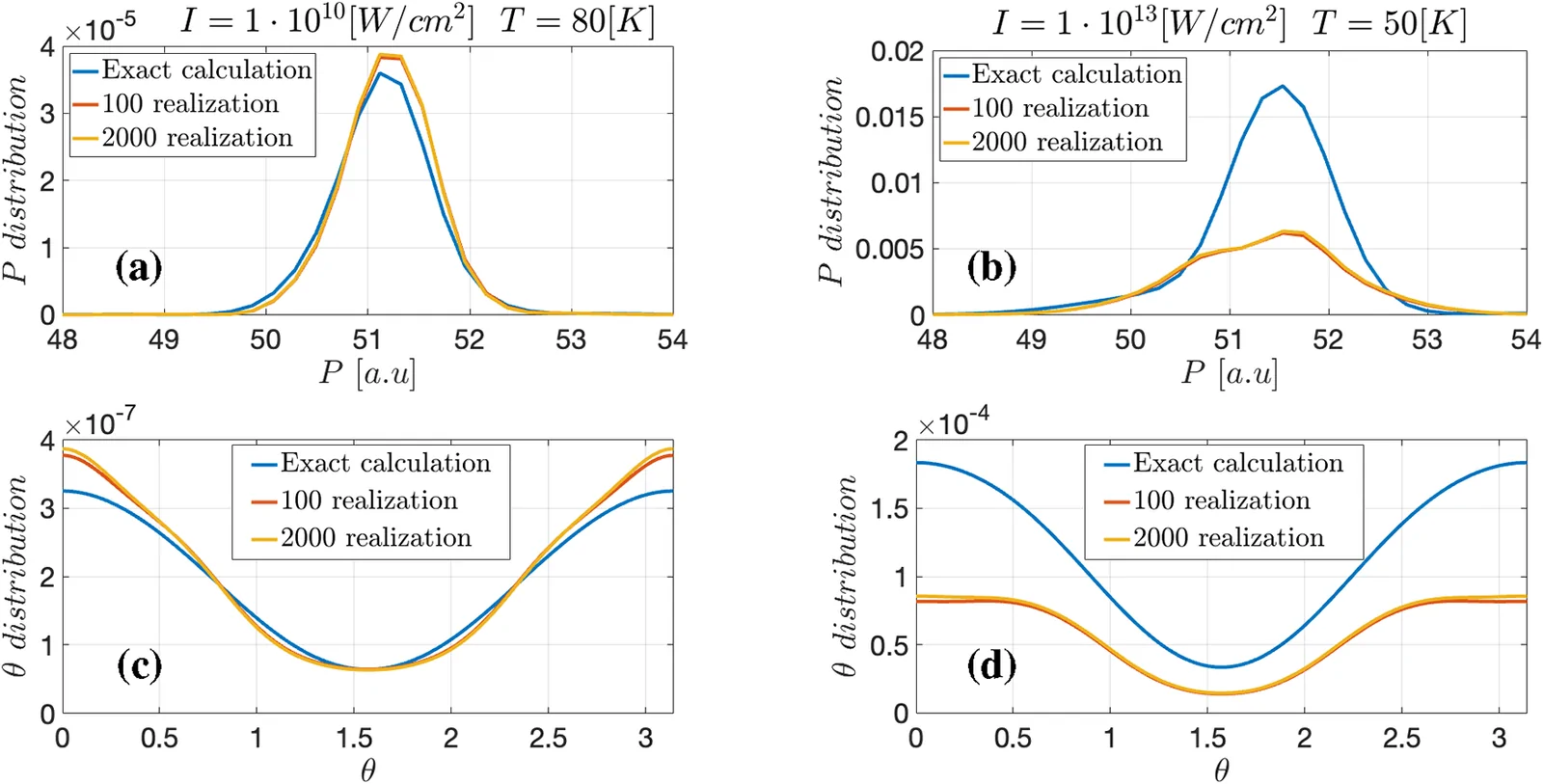
Upper panels: The outgoing momentum distribution as a function of the momentum in the parallel direction *P*_∥_. The exact calculation is compared to results obtained with different number of realizations. The panels correspond to low laser intensity (a) and high laser intensity (b). Lower panels: The outgoing momentum distribution as a function of the angle θ for the exact calculation and for different number of realizations. Low laser intensity, (c), and high laser intensity, (d). The figures sample a cut in the corresponding distribution in Figure 2, see text. Mind that while the angular axis span the whole range of values for θ, the presented result for the *P* axis is about 5% of the total grid, thus its resolution is significantly lower.

The distance between the distributions as a function of the number of realizations is calculated by the Wootters metric.^24^ The distance is calculated for the momentum distribution, *d*_W_^p^, and at the angular distribution, *d*_W_^θ^, defined by the following equations

13 $$D_{W}^{p}\left(K\right)=\frac{\pi }{2}cos^{-1}\left(\int P^{2}dP\sqrt{D_{rand}^{K}\left(P,\theta =0\right)}\cdot \sqrt{D_{exact}\left(P,\theta =0\right)}\right)$$

14 $$D_{W}^{\theta }\left(K\right)=\frac{\pi }{2}cos^{-1}\left(\int sin\left(\theta \right)d\theta \sqrt{D_{rand}^{K}\left(P=P_{mp},\theta \right)}\cdot \sqrt{D_{exact}\left(P=P_{mp},\theta \right)}\right)$$

We choose to calculate the distribution at the momentum direction, *d*_W_^p^, and the angular direction, *d*_W_^θ^, separately. For each calculation, we set one of the direction at the most probable value, θ = 0 and *p* = *p*_mp_. The distance was normalized so that its maximal value is 1 for nonoverlapping distributions. The distances between the distributions as a function of the number of realizations are present in Figure 4.

**Figure 4:**
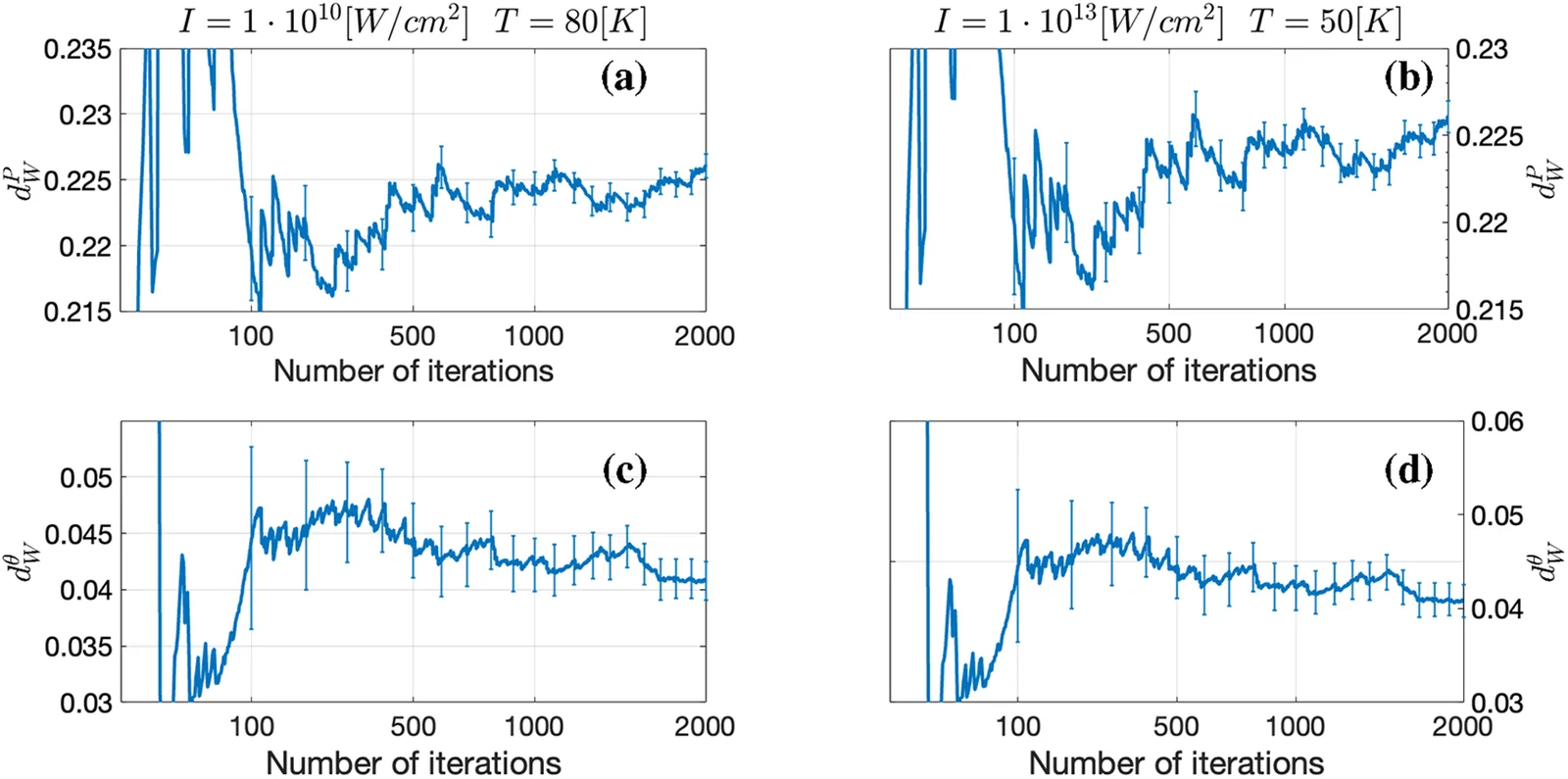
Distributions’ distance as a function of the number of realizations. Arranged similarly to Figure 3. (a, 4b) Display the distance between the momentum distributions comparing the random-phase method to the exact calculation, *d*_W_^p^, eq 13, while (c, 4d), present the distance of the angular distributions, *d*_W_^θ^, eq 14.

The convergence of the distribution is analyzed by the distance between the distributions at different laser intensities. An inspection of the distances in Figure 4, reveals that the distribution at low intensity, both in angular and momentum, is closer to the exact distributions. Furthermore, in both intensities, the momentum distribution is closer to the exact solution than the angular distribution. For an extremely narrow distribution such as that in our case, the distribution distance is sensitive to small differences and accounts for high levels of coherences. In contrast, observables that are more classical and contain a lower level of off-diagonal elements in the energy representation, such as the average kinetic energy and dissociation probability, are less sensitive to coherences and are much easier to capture with the random-phase method.

## Discussion and Conclusions

In this work, a computational method for the first-principle simulation of strong-field photodissociation and other time domain spectroscopy was developed. Simulations based on an exact solution of the time-dependent Schrödinger equation are expensive. Typical computational complexity scales as *N*_S_^3^, where *N*_S_ is the number of states involved in the process. Simulations of diatomic photodissociation processes at typical experimental conditions require a vast Hilbert space, of the order 10^6^.

To overcome this challenge, the RPW method has been employed. The phases of the initial states are randomly chosen and thermal averaged observables are obtained. The observables are converged to the directly calculated outcome after *K* realizations, where *K* ≪ *N*_S_. Previous studies on RPW^11,13,14^ showed that the observable convergence scales as $∼\frac{1}{\sqrt{K}}\cdot \frac{1}{\sqrt{N_{S}}}$ . Here, we show the required *K* is not universal: it depends strongly on the observable’s sensitivity to coherence.

As the RPW method relies on randomness of the relative phase between the states, it is unlikely that processes which are driven by high-order coherent interactions, e.g., multiphoton transitions, would be well approximated by the method. These types of processes are highly coherent and employ throughout the dynamics nondiagonal elements of the density matrix, i.e., coherences.

The dissociation probability P is an example of an observable in which the only contribution comes from diagonal elements of the density matrix in the energy representation. Hence, coherent interferences have low impact on such observables. Another case is the average kinetic energy, Δ*E*_*k*_^rel^, an observable that is less affected by the different interference pathways in the dynamics. At both intensities, it converges to the exact solution up to the experimental error of about 0.02 eV after only ∼ 200 realizations, which means acceleration of the calculation by a factor of ∼20 compared to the direct method, which propagates every initially occupied state separately. An explanation can be attributed to the property of the operators that correspond to these observables that are diagonal in the energy representation. Thus, they are less dependent on coherences and interferences.

Another observable that was explored is the momentum-space distribution of the dissociated fragments, which provides a visual approach to study the convergence. Our main result concerning this quantity is that its dominant features, such as the overall shape and peak position, are accurately recovered by the method, while fine details such as the peak height might be difficult to retrieve—at high intensity, the momentum-magnitude peak reaches only about a third of the exact value, and barely improves between 100 and 2000 realizations. This is the most coherence-sensitive observable we studied, and its slow convergence is the clearest illustration of our central finding. Unlike the dissociation probability and kinetic energy, this distribution carries substantial coherence between angular-momentum states, so it converges far more slowly. In addition, the convergence is better for the angular variable θ than for the magnitude of the momentum, due to the very different width of the distribution in these two directions. Within a single calculation, every observable shares the same *N*_S_ and the same *K*, yet at high intensity, the diagonal observables converge within 200 realizations while the momentum distribution does not converge after 2000. This within-run difference cannot be attributed to temperature, and therefore reflects coherence alone. Consistently, the split is pronounced in the high-intensity run, where coherence is largest, and negligible at low intensity, where both observable types converge fast. We remark that the common use of this tool in the experimental context is usually more quantitative—its main use is to assign the different fragments to their electronic channels. Therefore, our finding that the exact distribution is difficult to recover affects the applicability of the method only marginally.

The observed error leads us to conclude that the different observables converge differently. Each observable is sensitive to different properties of the photofragments state. The dynamics of the dissociation process include interferences. Adding random phases changes the interference pattern, leading to different outcomes. At high intensity, the process contains multiphoton transitions that induce rare events and interferences that lead to special states. Thus, adding a random phase leads to different events and interferences and to different final states.

The observable convergence is subject to the coherence and interference during the process. We note here that the choice of the wavefunction basis might be another factor that affects the observable convergence. However, upon changing the basis from the Wigner *D* matrices to a direct angular grid representation basis, we did not observe any significant shift in the convergence rate.

In conclusion, we constructed a first-principles model for time-domain spectroscopy for calculating experimental properties. The dynamical processes are computationally complex due to the large Hilbert space; thus, we used the RPW method to reduce the complexity. We studied the convergence properties of the product observables at two different laser intensities. The same method would apply to the dynamical hole left in the reactants.^25^ We conclude that the convergence depends on the sensitivity of the observable to the dynamic interferences that take place in the process. Thus, coherence-sensitive observables converge more slowly, and a high laser intensity worsens this by building stronger interference into the final states.
